# Voluntary modulation of saccadic peak velocity associated with individual differences in motivation

**DOI:** 10.1016/j.cortex.2018.12.001

**Published:** 2020-01

**Authors:** Kinan Muhammed, Edwin Dalmaijer, Sanjay Manohar, Masud Husain

**Affiliations:** a Nuffield Department of Clinical Neurosciences, University of Oxford, UK; b Department of Experimental Psychology, University of Oxford, UK

**Keywords:** Main sequence, Saccades, Motivation, Voluntary control, Incentives

## Abstract

Saccadic peak velocity increases in a stereotyped manner with the amplitude of eye movements. This relationship, known as the *main sequence*, has classically been considered to be fixed, although several recent studies have demonstrated that velocity can be modulated to some extent by external incentives. However, the ability to voluntarily control saccadic velocity and its association with motivation has yet to be investigated. Here, in three separate experimental paradigms, we measured the effects of incentivisation on saccadic velocity, reaction time and preparatory pupillary changes in 53 young healthy participants. In addition, the ability to voluntarily modulate saccadic velocity with and without incentivisation was assessed. Participants varied in their ability to increase and decrease the velocity of their saccades when instructed to do so. This effect correlated with motivation level across participants, and was further modulated by addition of monetary reward and avoidance of loss. The findings show that a degree of voluntary control of saccadic velocity is possible in some individuals, and that the ability to modulate peak velocity is associated with intrinsic levels of motivation.

## 1 Introduction

To create a high definition image of the world, humans direct the photoreceptor-dense fovea to objects of interest. Saccades are the mechanism by which this is performed. These fast, ballistic eye movements have classically been thought to follow a highly stereotyped relationship with the amplitude of eye movements. Velocity increases predictably with the amplitude of eye movements, a phenomenon referred to as the main sequence (Bahill, Clark, & Stark, 1975). This relationship is believed to reflect optimization of the trade-off between accuracy and duration of eye movements. Larger saccades require larger neural signals to increase speed and as a result generates extra neural noise, thereby reducing accuracy (Harris and Wolpert, 1998, Harris and Wolpert, 2006). This speed-accuracy trade-off is thought to explain the predictability of saccadic velocity.

However, there appears to be more flexibility in the main sequence than previously thought (Manohar, Muhammed, Fallon, & Husain, 2018). While saccadic peak velocity is considered to primarily be determined by saccadic amplitude, other factors are known to modulate it which have inter and intra-individual variations. Specifically, peak velocity is known to decrease as a function of time on task (App & Debus, 1998), mental fatigue (Bahill et al., 1981, Schmidt et al., 1979), sleep deprivation (Zils, Sprenger, Heide, Born, & Gais, 2005), and sedatives (Grace et al., 2010, Melichar et al., 2003). Saccadic velocity is also negatively correlated with cognitive load (Di Stasi et al., 2010, Di Stasi et al., 2011) and interestingly, eye movements are also faster when accompanied by an arm movement towards the same target in macaques (Snyder, Calton, Dickinson, & Lawrence, 2002) and humans (van Donkelaar, 1997). These findings suggest that multiple features are capable of producing deviation from the main sequence.

Expected value is also an important factor in advanced preparation of motor actions, such as saccadic eye movements (Dorris, 2007). Recent evidence demonstrates that saccades are not simply stereotyped ballistic movements, but can be modulated by incentives. For example, rewarded saccades have been shown to increase in velocity compared to unrewarded ones. This finding was first demonstrated in monkey studies. Saccadic properties towards targets that were rewarded with juice were compared to non-rewarded targets. Under these circumstances, the expectation of reward significantly increased saccadic velocity (Chen et al., 2013, Takikawa et al., 2002). Further investigations confirmed these findings, including eye tracking tasks performed in humans; these revealed modulation of saccadic peak velocity when monetary rewards were offered (Chen et al., 2014, Manohar et al., 2015, Manohar and Husain, 2016). There have since been numerous theoretical and experimental papers that have considered saccade vigour and its dependence on reward, particularly in humans. Shadmehr et al. initially provided a theoretical basis for saccade velocity modulation, and linked it to disease states and psychiatric conditions that affect reward encoding in the brain (Shadmehr, Smith, & Krakauer, 2010). Subsequently, the modulation of saccadic vigour has also been associated with individual aspects of decision-making (Choi et al., 2014, Haith et al., 2012) and during the act of reward comparison (Reppert, Lempert, Glimcher, & Shadmehr, 2015). Most recently, a study suggested that the vigour of saccades and other movements also appear to be a trait-like feature of individuality (Reppert et al., 2018).

The invigoration of saccadic velocity has been associated with dopamine-reward signals from the basal ganglia studied in human and animal models. This subcortical region is involved in the control of eye-movements (Hikosaka, Takikawa, & Kawagoe, 2000) and also in reward based decision making (Hikosaka, Nakamura, & Nakahara, 2006). This allows for the potential study of incentive processing and motivation through the assessment of saccadic vigour. The ventral pallidum and ventral striatum are basal ganglia structures which play key roles in processing motivational information, projecting to and receiving inputs from subcortical and cortical regions implicated in the motivational control of behaviour (Haber & Knutson, 2010). Indeed, neuronal recordings from monkey ventral pallidum revealed that, depending on the amount of expected reward, neurons either increased or decreased their activity and this appears linked to changes in saccadic velocity (Tachibana & Hikosaka, 2012). The neurotransmitter dopamine has been implicated in this process, demonstrated through MPTP (1-methyl-4-phenyl-1,2,3,6-tetrahydropyridine) induced lesions in the caudate nucleus of primates (Kori et al., 1995) and in humans using Parkinson's disease patients with dopamine depletion (Manohar et al., 2015).

Dysfunction of reward processing in the brain has been associated with difficulties in goal directed behaviour and linked to motivational deficits. These include disorders such as pathological apathy in patients with neurological diseases, for example those with Parkinson's disease, small vessel disease or following types of stroke such as subarachnoid haemorrhage (Heron et al., 2018, Manohar and Husain, 2016, Mattox et al., 2006). Likewise, dysfunctional responses to punishment may be equally important to behavioural outcomes and might also contribute to motivation disorders associated with reduced activity or vigour. In a recent study, the prospect of penalty increased saccadic vigour only when it was dependant on performance (Manohar & Husain, 2016) again suggesting an association with motivation. However, previous investigations have not examined within the healthy population, whether individual differences in level of motivation affect how external incentives modulate the voluntary control of saccadic velocity.

In addition to saccadic velocity, physiological responses to incentives indexed by autonomic modulation of pupil size has been shown to aid our understanding of goal directed behaviour. This autonomic response has been demonstrated in pupil dilatation when anticipating rewards and punishment (Manohar & Husain, 2016). Moreover, pupil responses to incentives appear strongly correlated with motivation level in patient populations such as Parkinson's disease and are further modulated by dopamine (Muhammed et al., 2016). Thus, patients with apathy have blunted pupil responses to reward, indicative of reduced reward sensitivity in these individuals; while the addition of dopaminergic medication enhances pupillary dilatation in anticipation of rewards.

Although saccadic velocity has now been shown to increase with incentives, to the best of our knowledge it remains to be established whether saccadic velocity can be controlled voluntarily. Here we attempted to examine people's ability to do this and the relationship of performance with an independent questionnaire measure of motivation level. Three studies were conducted to assess if it was possible to voluntary modulate saccadic peak velocity, thereby breaking the stereotypical relationship of the main sequence. The addition of reward and loss was also explored and the effect on pupil dilation and other oculomotor properties examined. We hypothesised that it might be possible to voluntarily control saccadic velocity to some extent, that incentives would increase this further and that the ability to do so might depend on motivation level. The first experiment assessed the ability of participants to modulate saccadic velocity voluntarily with no incentivisation, the second examined this ability with monetary reward, and the third in loss avoidance. In order to assess associations with motivation, we examined voluntary rather than reflexive saccades as this may be a more accurate representation of subjects internal motivation level.

## 2 Methods

### 2.1 Participants

The study was approved by the local ethics committee and written consent was obtained. Participants were informed that they would be paid according to performance on the task, a minimum of £8 and maximum of £12 was paid. 53 young healthy participants were recruited in total over three experiments using online adverts placed in the Oxfordshire area.

All participants completed the Extended Lille Apathy Rating Scale (LARS-e) questionnaire (Ang et al., 2017, Bonnelle et al., 2015). This is an adapted 51-point item questionnaire designed to measure apathy levels in non-patient populations, which assess different domains of apathy based on a participant's view of his/her life over the previous two weeks. The LARS-e is based on The Lille Apathy Rating Scale (LARS) (Sockeel, 2006) which was developed for the assessment of clinical apathy in patients groups. It is comprised of 4 subdomains, each linked with different domains of motivation, including: reduced intellectual curiosity, emotional indifference, reduced action initiation, and lack of self-awareness.

### 2.2 Study One | voluntary control of peak saccadic velocity

18 young healthy subjects were tested while eye position and pupil diameter was monitored using an infrared eye tracker (Eyelink 1000, SR Research). The average age of participants was 30 years (± 8 years), 10 participants were male and all had normal or corrected to normal vision. Participants were first instructed to perform 15 saccades from a fixation cross located on the left side of the screen to a target that appeared at a constant eccentricity of 22.5° to the right. This was used to calculate the average baseline saccadic velocity for each individual. Following this, the experimental paradigm began.

**Fig. 1:**
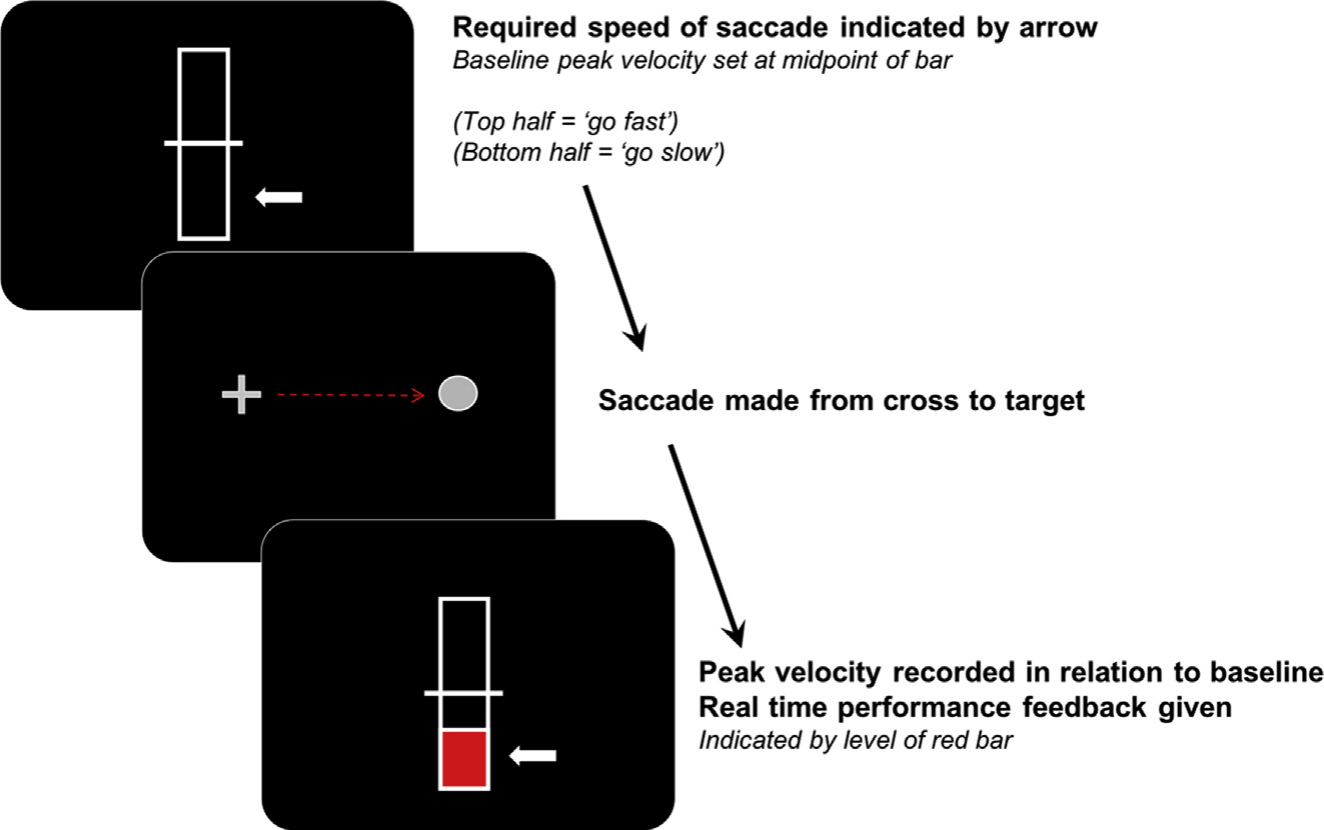
**Experimental paradigm to study voluntary control of saccadic velocity.** Baseline saccadic velocity was set to the midpoint of the bar. The required velocity was indicated at the beginning of each trial. Slow velocity saccade trials had a white arrow pointing to the bottom half of the marker while fast velocity saccades were indicated by the arrow pointing to the top half of the bar. Participants were required to modulate their saccadic speed appropriately. After a saccade was made, real time feedback was given on performance by the level of a red bar, this was based on the difference in velocity from each participant's baseline.

There were two trial types: slow or fast. Participants were instructed to make either slow or fast velocity saccades respectively. The trial type was depicted by an arrow pointing at the top or bottom of a vertical bar at the start of each trial (Fig. 1). If the arrow pointed to the top half of the bar this indicated a fast saccade was required on that trial; if the arrow pointed at the bottom half, a slow saccade was required. Saccades (slow or fast) were made between a fixation cross and a target held at a constant 22.5°. The fixation cross was 11.25° to the left of the centre of the screen, and the target 11.25° to the right of the centre of the screen. Slow or fast trials were intermixed and there were 100 saccades per condition in total. Online feedback based on real time peak velocity was given after each trial to inform participants of their performance. This was indicated by the level of a red bar which was then displayed on the screen (see Fig. 1), the feedback was provided in a continuous fashion, as a function of the online measurement of saccadic peak velocity. Saccades were always made from left to right, after completing the saccade, the feedback would be displayed and then the next trial would start.

### 2.3 Study Two | effects of monetary rewards on voluntary control of saccadic velocity

A new sample of participants were recruited in this study designed to examine whether monetary incentives modulate voluntary control of saccadic velocity. 19 healthy people were tested, 10 male; mean age 26 years (± 5 years). To calculate baseline saccadic velocity and avoid any fatigue effects, participants were first instructed to make 10 fast saccades from a fixation cross to a target at 22.5° eccentricity. They were then instructed to make 10 slow saccades. The average fast and slow saccadic velocities were then used to calculate baseline velocities for the two conditions.

On subsequent trials during the experiment, if a fast saccade was required then this was compared to the slow trials’ baseline. If the saccade was faster than the average slow baseline velocity then this was counted as a correctly modulated fast saccade. The opposite was true for slow saccades. This design allowed an adaptive baseline to be established throughout the experiment, with the preceding 20 trials used to adapt the fast and slow baselines to take into account any effects of fatigue over the course of the experiment.

**Fig. 2:**
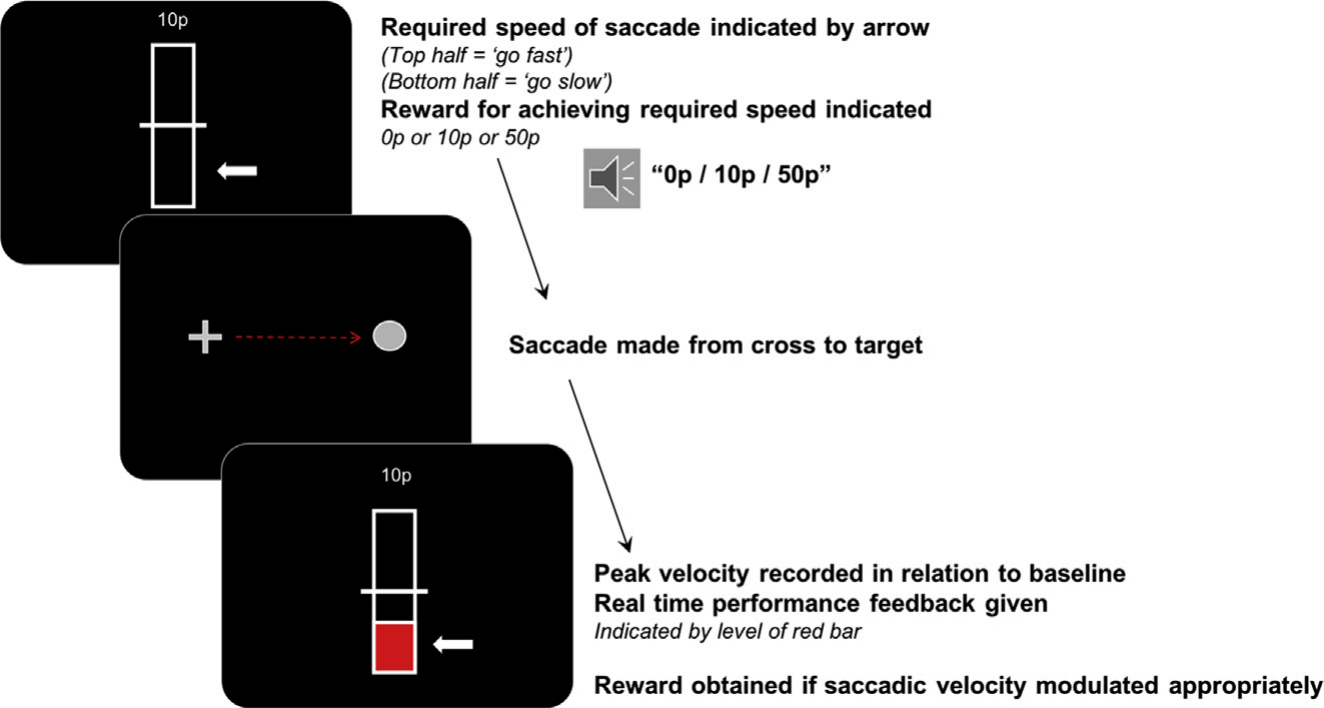
**Experimental paradigm to examine the effects of monetary rewards.** Baseline saccadic velocity was set to the midpoint of the bar. This was based on the average of the proceeding 20 trials and was set as the mean slow velocity when a fast trial was being performed and the mean fast velocity during a slow saccade trial. The required velocity was then indicated at the beginning of each trial. Slow velocity saccade trials were depicted by a white arrow pointing to the bottom half of the marker and fast velocity saccades by an arrow pointing to the top half of the bar. Simultaneously the reward at stake for correct performance was delivered over the loud speakers and as text on the screen. Participants were required to modulate their saccadic velocity appropriately. After a saccade was made, real time feedback was given on performance by the level of a red bar and the amount of reward obtained on that trial was also presented.

Monetary rewards were included in this experiment as an added incentive to modulate saccadic velocity (Fig. 2). One of three reward levels (0p, 10p or 50p) was presented via a loud speaker and as text on the screen at the start of each trial. Simultaneously with the reward cue, a white arrow indicated the fast or slow saccade trial type. After participants made a saccade, they received all or none of the available reward depending on whether they had been able to modulate saccadic velocity as instructed. Participants were told that they would be paid for their time depending on their performance during the experiment. Fast and slow trials were intermixed and there were 150 saccades per condition in total. Real time feedback was indicated by a red bar at the end of each trial, as in the previous study.

### 2.4 Study Three | effects of monetary losses on voluntary control of saccadic velocity

**Fig. 3:**
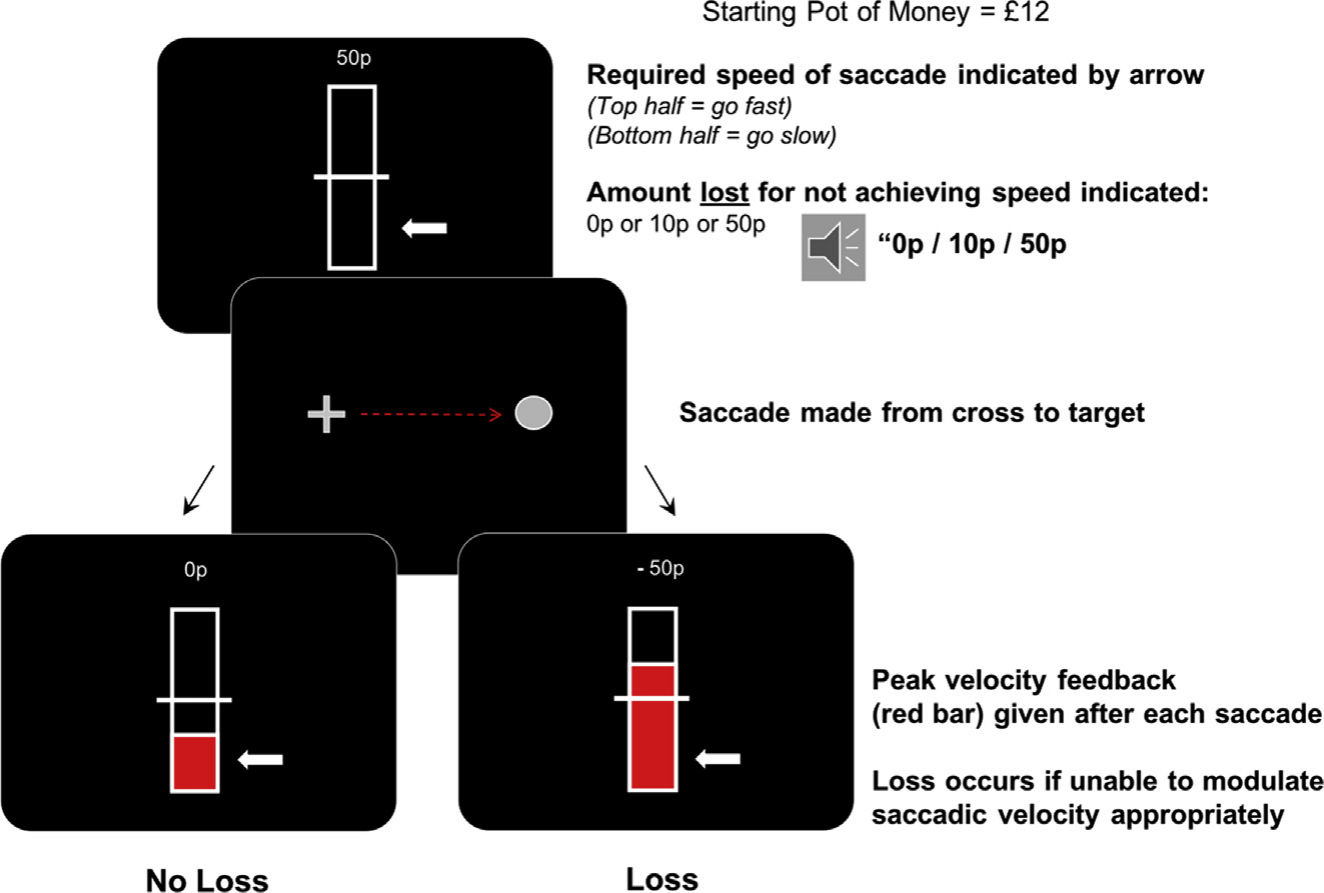
**Experimental paradigm to examine the effects of monetary losses.** Baseline saccadic velocity was set to the midpoint of the bar, based on the average of the proceeding 20 trials and was set as the average slow velocity when a fast trial was being performed and the average fast velocity during a slow saccade trial. The required velocity was indicated at the beginning of each trial, with an arrow pointing to the top or bottom, as in the previous experiments. Simultaneously the potential loss at stake for incorrect performance was presented over the loud speakers and as text on the screen. After a saccade was made, real time feedback was given on performance by the level of a red bar and the amount of money lost on that trial was indicated. Money was lost if participants were unable to correctly modulate saccadic velocity.

A different group of 16 healthy participants were tested (12 male; mean age 25.3 years ± 5.4) to examine whether monetary losses affected voluntary control of saccadic velocity. The method employed in Study Two was used in order to establish fast and slow baseline velocities for each person. Similarly, adaptive modulation of baseline velocity was computed using the preceding 20 trials. However, in this experiment, instead of rewards being offered to perform the required fast or slow trial type, the amount of money that would be lost if the required velocity was not performed correctly was now presented at the start of each trial. This was presented via the loud speakers and also as text at the start of the trial. Again, trial type was simultaneously presented with the loss cue and was depicted by the position of the white arrow (Fig. 3). If the performed saccade did not match the required fast or slow trial type then participants lost the amount of money initially displayed. If they were correct then they did not lose any money and the next trial would begin. Participants were told at the start of the experiment that they would begin with £12 and, depending on performance, they would have money deducted from their final payment.

All three experiments were saccadic overlap tasks, the fixation cross remained on screen after the target appeared allowing participants to initiate movements at their own pace. The time course was as follows: Fixation cross appeared at time 0 msec, this was then followed by a random interval of between 750 msec and 1250 msec to avoid anticipatory saccades, then the target appeared while the fixation cross remained on screen throughout. The baseline trials for all experiments were measured using the same time course as the main experimental paradigms. For further details on participant payment calculation please see Supplementary materials.

### 2.5 Eye tracker data recording and analysis

Eye tracking was performed in a dimly lit room 60 cm in front of a 21” CRT (1024 × 768 pixels; 100 Hz refresh). Stimuli were presented on a Windows computer running Python, using PyGaze (Dalmaijer, Mathôt, & Van der Stigchel, 2014) and PsychoPy (Peirce, 2007). The frame-mounted infrared tracker (Eyelink 1000, SR Research) monitored left eye position and sampled at 1 kHz. A 5-point calibration was performed at the start of each experiment. These included the display centre and across the 4 midpoints of the screen edge in a cross configuration, these points were 10% from the screen edge, allowing for a horizontal and vertical calibration.

Eye movements were measured online using PyGaze's built-in velocity-based algorithm to detect saccade onsets and offsets. Peak saccadic velocity was computed in real time as the maximum velocity recorded during the saccade and was directly presented as feedback on performance. An eye movement was classified as a valid saccade if it started within 2° from the fixation cross, and landed within a 5° radius from target centre. This detection method was only used for the online delivery of feedback. All further offline analysis, including velocities and amplitudes, used standard Eyelink criteria for the saccadic detection which also use a velocity and acceleration criterion. Peak velocity was estimated from 3 msec windows during the saccade. Eye movement analysis was carried out using custom Matlab code and statistical analysis was performed using SPSS. See Supplementary materials for further details on online and offline saccadic criteria and analysis.

Although the target eccentricity was fixed in all three studies, each saccade varied in absolute amplitude, so it is important to take this into account in any analysis. Residual saccadic velocity was calculated in order to factor out any such effects of the main sequence: amplitude of saccades were regressed out from the measured peak velocities. The regression-based model used to control for saccade amplitude was a linear model and velocity was not constrained to be zero at zero amplitude:

Velocity_i=V_0+kAmplitude_i

Where velocity_i and amplitude_i are the peak saccade velocity and saccade amplitude on an individual trial, V_0 and k are the fitted intercept and slope.

Saccadic accuracy was defined as the mean SD of saccadic amplitudes for each condition. Using saccadic variability as a measure of accuracy accounts both for any changes in starting position due to eye tracker drift as well as variability in the end point error of each saccade.

Pupil dilation was measured in Eyelink units, which are arbitrary units but stable within a participant and provide a reliable measure of pupil size. Recordings were time locked to the target onset and normalised using a 200 msec baseline subtraction for each trial. Pupil traces lost due to blinks were interpolated. A moving average smoothing window of 100 msec was applied to the final recordings. Average pupil recordings were taken over a 700 msec time window between 500 msec and 1200 msec with the reference time point at cue onset (0 msec).

## 3 Results

### 3.1 Study One | voluntary control of peak saccadic velocity

#### 3.1.1 Saccadic eye movement velocity can be voluntarily modulated

**Fig. 4:**
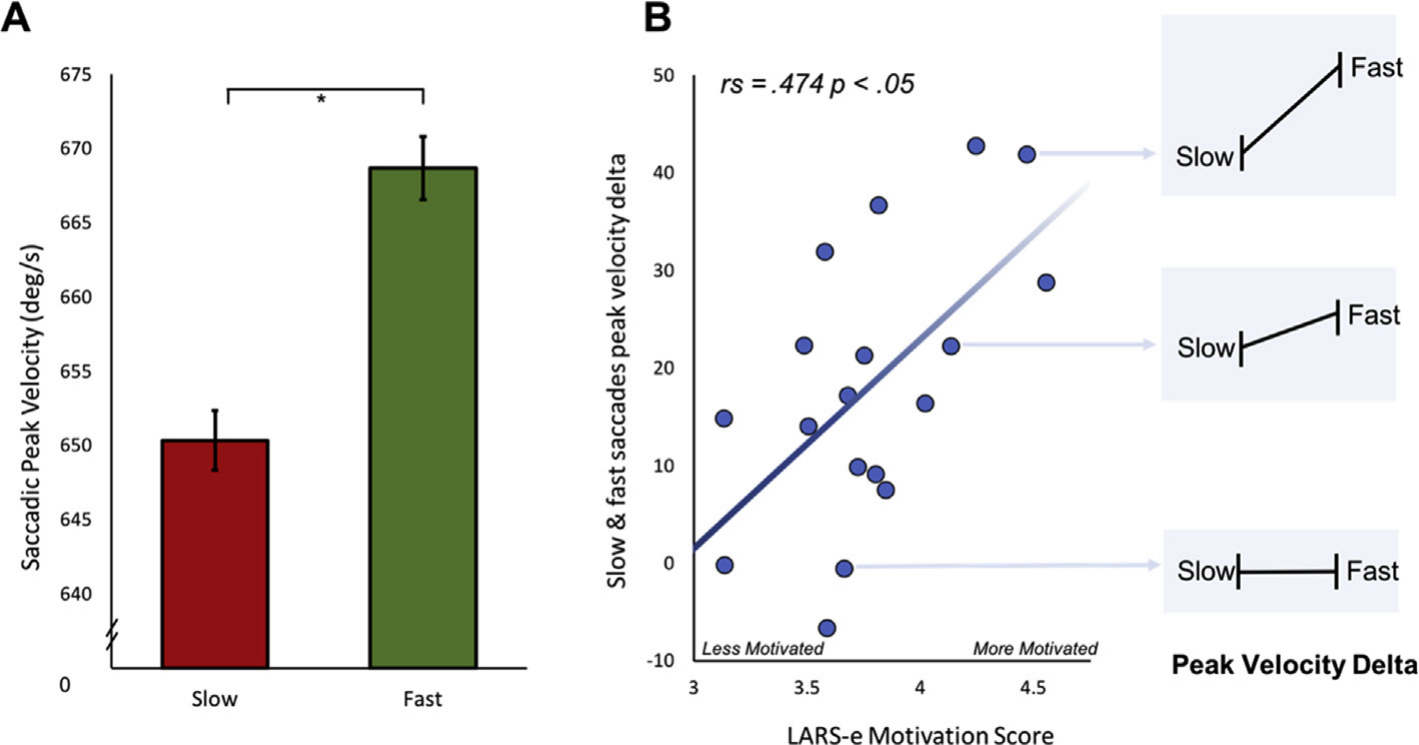
**Saccadic velocity modulation and association with motivation. A.** Average saccadic peak velocity for slow (red) and fast (green) saccade trial types. Error bars are average within subject standard error of the mean. **B.** Participants' ability to modulate saccade peak velocity (calculated as the average difference between fast and slow trial types for each participant) positively correlated with motivation level. Those with greater ability to modulate saccadic velocity had higher motivation score on the LARS-e. The panel to the right depicts different degrees of modulation for three different people, for illustrative purposes.

A significant difference was found when comparing average saccadic peak velocity between fast and slow trial types. A paired *t*-test between fast (M = 668.7 deg s^−1^, SEM = 23.7) and slow (M = 650 deg s^−1^, SEM = 22.8) conditions demonstrated that individuals have the ability to voluntarily alter the velocity of their saccadic eye movements [t (17) = -5.46, *p* < .0001; Fig. 4A].

#### 3.1.2 The ability to modulate saccadic peak velocity correlated with motivation level

There was a significant correlation between the ability to modulate saccadic peak velocity and the degree of apathy scored on the LARS-e questionnaire. Spearman's rank correlation comparing the difference in mean peak velocity residuals between fast and slow conditions and participants total LARS-e score was significantly correlated (rs = .474, *p* < .05; Fig. 4B). Thus more motivated individuals had greater ability to modulate their peak saccadic velocity.

**Fig. 5:**
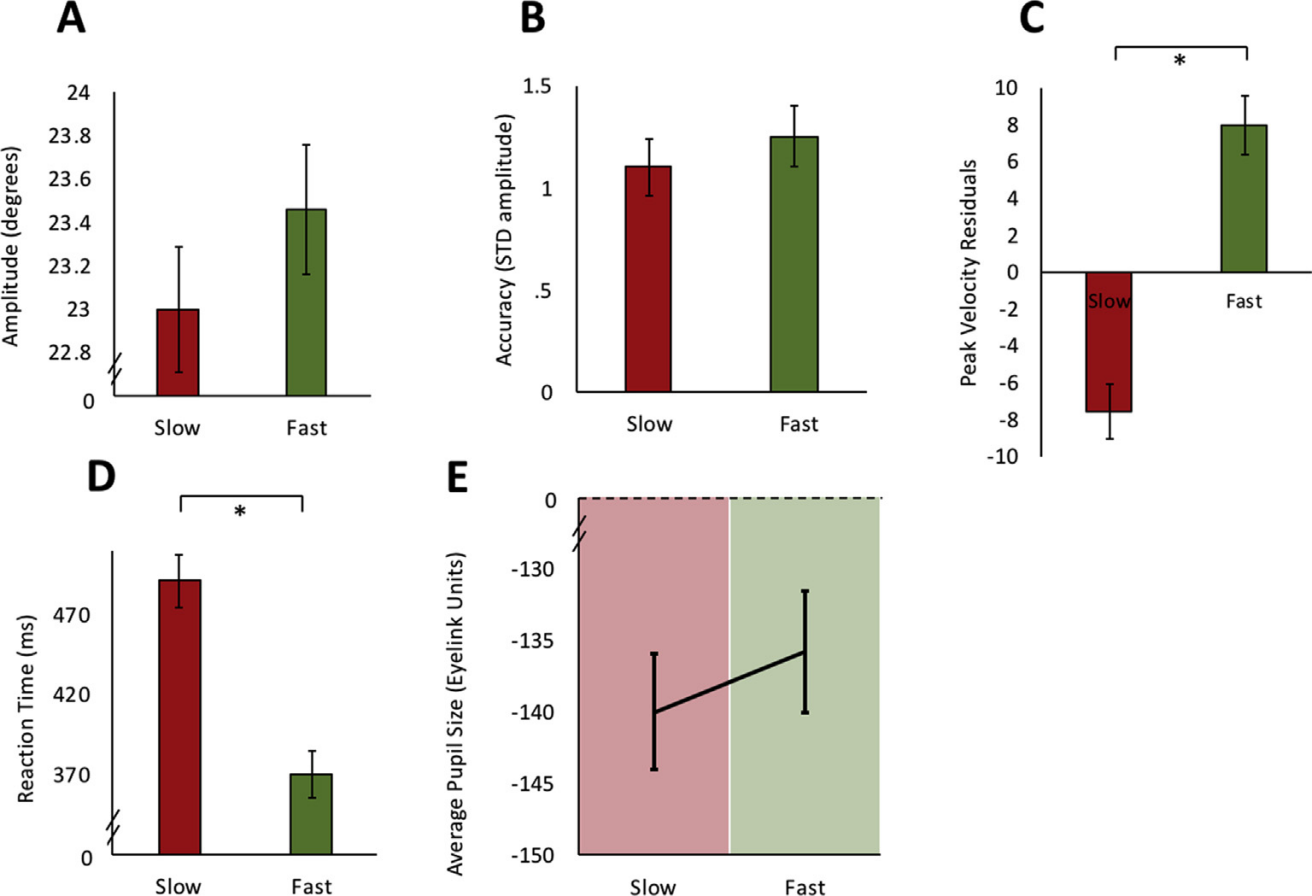
**Oculomotor and pupil properties in slow and fast saccadic velocity conditions. A.** There was no significant difference between mean amplitude of saccades for each condition. **B.** No significant difference in variability was demonstrated between fast and slow conditions. Variability was used as an assessment of accuracy, calculated as SD of mean saccadic amplitude. **C.** Average residual velocity after amplitude is regressed out from saccadic peak velocity on a trial by trial basis. Fast trials remained significantly faster than slow trials even after accounting for any differences in amplitude. **D.** Reaction times were significantly longer when required to make slower saccades. **E.** There was no significant difference in pupil size (in Eyelink arbitrary units) prior to saccade. All error bars are within subject standard errors of the mean.

The main sequence demonstrates that saccades of larger amplitudes have faster peak velocities (Bahill et al., 1975). Therefore it was also important to check that differences in saccadic amplitude for the two different trial types (fast and slow saccades) did not account for our main finding. Comparisons between the amplitudes in the fast (M = 23.46 deg, SEM = .66) and slow conditions (M = 22.99 deg, SEM = .68) showed no significant differences [paired *t*-test: t (17) = -.717, *p* = .483; Fig. 5A].

To assess if accuracy was different between the fast and slow trial types, the variability of saccades using the mean SD of amplitudes was calculated. Although there was some variability in the amplitude of saccades made, participants did not demonstrate any significant difference in variability between fast (M = 1.26 deg, SEM = .15) and slow (M = 1.10 deg, SEM = .14) saccade trials [paired *t*-test: t (17) = -1.4, *p* = .18; Fig. 5B]. Variability accounted for differences in both starting position drift and also end accuracy of the saccades made.

In spite of there being no differences in saccadic variability or the average amplitude of saccades made for each condition, and in order to carry out a stringent analysis and strengthen the confidence in the results, any differences in amplitude following each saccade made by an individual was regressed out of the saccadic peak velocity for that trial. The peak velocity residual represents a value which can only be accounted for by saccadic velocity and removes any linear differences which might be secondary to the effects of the main sequence, i.e., due to differences in amplitude. Even after this analysis there was still a strongly significant difference between residual velocities in the fast (M = 8 deg s^−1^, SEM = 1.65) and slow (M = −7.6 deg s^−1^, SEM = 1.54) conditions [paired *t*-test: t (17) = -4.88, *p* < .001; Fig. 5C]. The residual velocities quantified main-sequence violations: they are the difference between the measured saccadic peak velocity, and the saccadic peak velocity that one would have predicted based on the corresponding saccadic amplitude. A positive residual velocity represents an underestimation of a saccade's peak velocity based solely on that saccades amplitude, whereas a negative residual velocity represents an overestimation of a saccade's peak velocity based on its amplitude. Positive residual velocities were found for the fast condition, representing an underestimation of saccadic peak velocity through the saccadic amplitude on the basis of the main sequence. The opposite held true for the slow condition. This suggested that participants were able to modulate their saccadic peak velocity different to that predicted from the main sequence. These results further strengthen the main finding that it is possible to voluntarily modulate saccadic peak velocity. There was also a significant correlation between LARS-e and the residual peak velocities, rs = .515, *p* < .03, this correlation was even stronger than when amplitude hadn't been regressed out (Fig. 4B). Reaction times were also significantly shorter in the fast condition compared to the slow. [paired *t*-test: t (17) = 4.3, *p* < .001; Fig. 5D].

#### 3.1.3 Pupillary differences in preparation for making fast and slow saccades

Paired *t*-tests were performed on the average pupillary change over the 500–1200 msec time epoch after the fixation cross onset, during the preparatory phase to make a saccade. No differences in pupillary size were found before making a fast (M = −135.73 Eyelink units, SEM = 88.1) or a slow (M = −139.94 Eyelink units, SEM = 82.44) saccade [paired *t*-test: t (17) = -.4.85, *p* = .63 Fig. 5E].

### 3.2 Study Two | effects of monetary rewards on voluntary control of saccadic velocity

#### 3.2.1 Rewards invigorate saccadic peak velocity

**Fig. 6:**
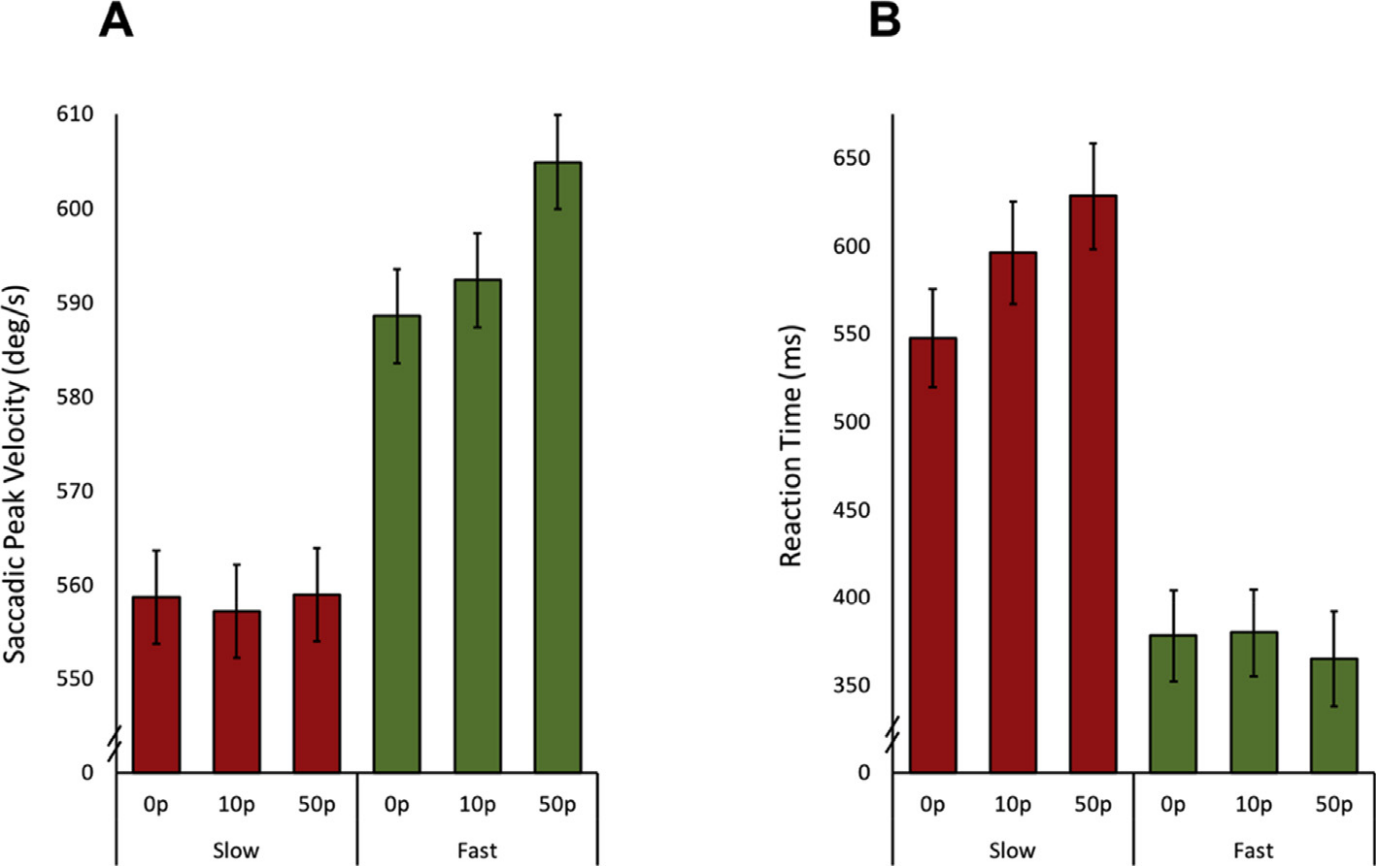
**Effect of reward on slow and fast saccadic velocity conditions. A.** Average saccadic velocity in the fast and slow conditions for different levels of incentive. There was a significant difference in overall speeds between the two groups, however incentive only invigorated the velocity of saccades in the fast condition trials. **B.** Reaction times for slow saccadic velocity trials were prolonged, with rewards having a modulatory effect, increasing reaction times further for larger rewards. In the fast condition trials, no significant differences between reward conditions were observed.

A repeated measures ANOVA was performed to investigate the effect of reward on voluntary control of saccadic velocity. Peak velocity residuals were used as the metric of comparison to ensure no effects were attributed to differences in amplitude in any of the conditions. The analysis was also performed on absolute velocity which produced the same findings. Although peak velocity residual were reported statistically, absolute peak velocities are depicted in the graphs for ease of understanding and clarity. To begin with, the ability to modulate saccadic velocity shown in Study One was replicated by a main effect of trial type (slow *vs* fast), F (1,18) = 43.56, *p* < .00001; Fig. 6A. Secondly, the reward offered (0p *vs* 10p *vs* 50p) on each trial had a significant effect on saccadic velocity [main effect of reward F (2,36) = 9.08, *p* < .001]. However, higher potential rewards invigorated saccadic peak velocities more when performing faster saccades [reward × velocity interaction F (2,36) = 5.44, *p* < .01].

Bonferroni corrected comparisons breaking down the effects of rewards in the two velocity conditions showed that there was no significant effect of reward when going slow (0p *vs* 10p, 10p *vs* 50p and 0p *vs* 50p, all *p* = 1.0). In contrast, in the fast condition, significant differences in velocity between the 0p and 50p condition and the 10p and 50p condition were present (0p *vs* 10p *p* = .75, 10p *vs* 50p *p* = .01, 0p *vs* 50p *p* < .01; Fig. 6A). In this experiment, which included the addition of rewards, there were no significant correlations between saccadic peak velocity or the differences between slow and fast peak velocities and the LARS-e.

#### 3.2.2 Changes in saccadic velocity are not due to amplitude differences or saccadic variability

To further clarify that differences were not due to saccadic amplitude changes, repeated measures ANOVA on average amplitude and amplitude variability (SD of amplitude) for each subject in all conditions were performed. There were no significant differences between overall amplitudes [main effect of velocity condition fast *vs* slow F (1,18) = 2.54, *p* = .13; main effect of reward F (2,36) = 1.14, *p* = .87, reward × velocity interaction F (2,36) = 1.81, *p* = .18]. Further, there were also no significant differences in amplitude variability either [main effect of velocity condition F (1,18) = .510, *p* = .48; main effect of reward F (2,36) = .28, *p* = .76; reward × speed interaction F (2,36) = 2.54, *p* = .1].

#### 3.2.3 Trial to trial effects

Trials in experiment two were split according to the previous trial's outcome. These could be error outcomes, which is when participants failed to correctly modulate their saccade for any of the three reward levels of 0p, 10p or 50p. They could also be non-error outcomes when the previous trial was a correct modulation of saccadic peak velocity for either 0p, 10p or 50p rewards. This was for both slow and fast saccade trial types. A 4 × 2 ANOVA was used to test the effect of the previous trial's outcome. Previous studies have studied reward-history effects on behavioural vigour (Griffiths & Beierholm, 2017). One might therefore expect that after a reward was obtained, saccade velocity may be higher on the next trial due to increased vigour effects. Alternatively reward history effects could arise simply if speed on the previous trial is correlated with speed on the current trial. To test these possibilities, we compared peak velocity on trials preceded by an incentive which was successfully obtained, and also when incentives were not obtained due to incorrect velocity modulation, this was also performed on trials preceded by a 'fast' or 'slow' trial type instruction. There was a main effect of the speed cue trial type [F (1,144) = 9.287, *p* < .001] but no effect of previous trial reward [F (3,144) = .014, *p* = .998] nor interaction [F (3,144) = .016, *p* = .997]. This indicated that there was no effect of the previous trial on ability to successfully modulate saccadic peak velocity. See Figure S3A in supplementary materials.

Trials were also split according to whether the instruction on the previous trial was to go fast or slow. There was no effect of the previous speed cue trial type: A 2 × 2 ANOVA was performed to test the effect of the speed cue on the previous trial, including previous and current trial types as factors. There was no effect of the speed cue trial type on the previous trial [F (1,72) = .526, *p* = .471], and no interaction with the current trial [F (1,72) = .215, *p* = .644]. See Figure S3B. The same trial to trial findings for rewards reported in experiment two were also true for the loss conditions in experiment three.

### 3.3 Reaction times increased by reward when making slow saccades

Significant differences in the reaction time data were found between velocity and reward levels. Using Greenhouse Geisser corrected repeated measures ANOVA to account for non-sphericity, a main effect of velocity was present, with the fast saccade condition also having faster reaction times, replicating the results from Study One [main effect of velocity, F (1,18) = 21.56, *p* < .001]. A significant interaction was also found: slow saccades demonstrated longer latencies for bigger rewards on offer, whereas the fast saccades had no latency differences between reward levels [velocity × reward interaction F (1.41,25.40) = 8.83, *p* < .01; Fig. 6B]. Bonferroni corrected pairwise comparisons in the fast condition showed no differences between reward levels (0p *vs* 10p *p* = 1.0, 10p *vs* 50p *p* = .97, 0p *vs* 50p *p* = 1.0). However, in the slow condition there was a significant difference between 0p and 50p reward (*p* = .046). The other comparisons did not reach significance (0p *vs* 10p *p* = .23, 10p *vs* 50p *p* = .08).

### 3.4 Pupillary differences during preparation for fast and slow saccades in anticipation of incentives

Using the same time epoch as in Study One, the average pupil change during the preparation period before making a saccade to obtain a reward was examined (500–1200 after the fixation cross appeared). There was a main effect of reward on pupil size and an interaction between reward on offer and the subsequent saccadic velocity (slow *vs* fast) that was being prepared for [main effect of reward F (2,36) = 14.12, *p* < .0001; reward x velocity condition interaction F (2,36) = 11.02, *p* < .001, main effect of velocity condition F (1,18) = 1.41, *p* = .25].

**Fig. 7:**
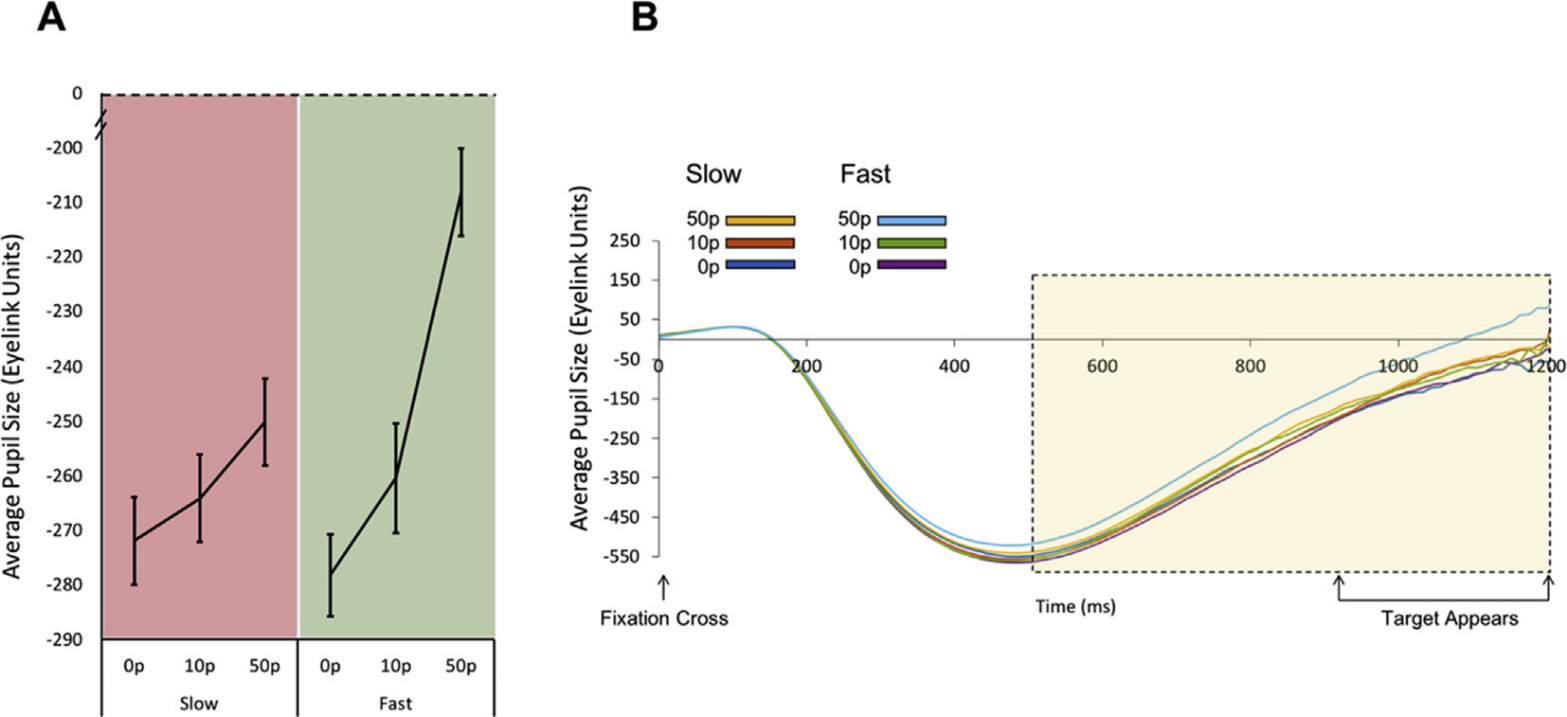
**Pupillary response to rewarding incentives in the slow and fast velocity conditions. A.** Average pupil size (in Eyelink units) prior to making a slow (red) or fast (green) saccade. Fast saccades had a greater change in pupil size for increasing reward levels compared to slower saccades. Average taken over a 700 msec time epoch (dashed area in Fig. 7B). **B.** Average pupil change over time from the start of the trial to when the target appeared. The target appeared at random time points between 750 msec and 1200 msec to avoid anticipatory saccades. The largest 50p incentive in the fast trial condition had the greatest pupil change compared to the other incentives including those in the slow condition. Yellow dashed area indicates the time period of interest from 500 msec to 1200 msec used to estimate average pupil changes for statistical comparisons.

When greater reward was on offer there was increased pupillary dilation over time (Fig. 7B) and also an interaction showing that bigger modulation of pupillary size by reward occurs when generating faster saccadic movements (Fig. 7A). Bonferroni corrected pairwise comparisons in the fast condition showed significant differences in pupil size between the 10p versus 50p condition (*p* = .004), and also in the 0p versus 50p reward level comparison (*p* < .0001); but no significant difference was observed between 0p versus 10p (*p* = .19). In comparison, in the slow condition, there was only a marginal difference in pupil size between the 0p versus 50p reward level (*p* = .045). No other significant difference was observed between 0p versus 10p (*p* = 1.0) and 10p versus 50p (*p* = .602).

### 3.5 Study Three | effects of monetary losses on voluntary control of saccadic velocity

#### 3.5.1 Loss did not modulate saccadic peak velocity

**Fig. 8:**
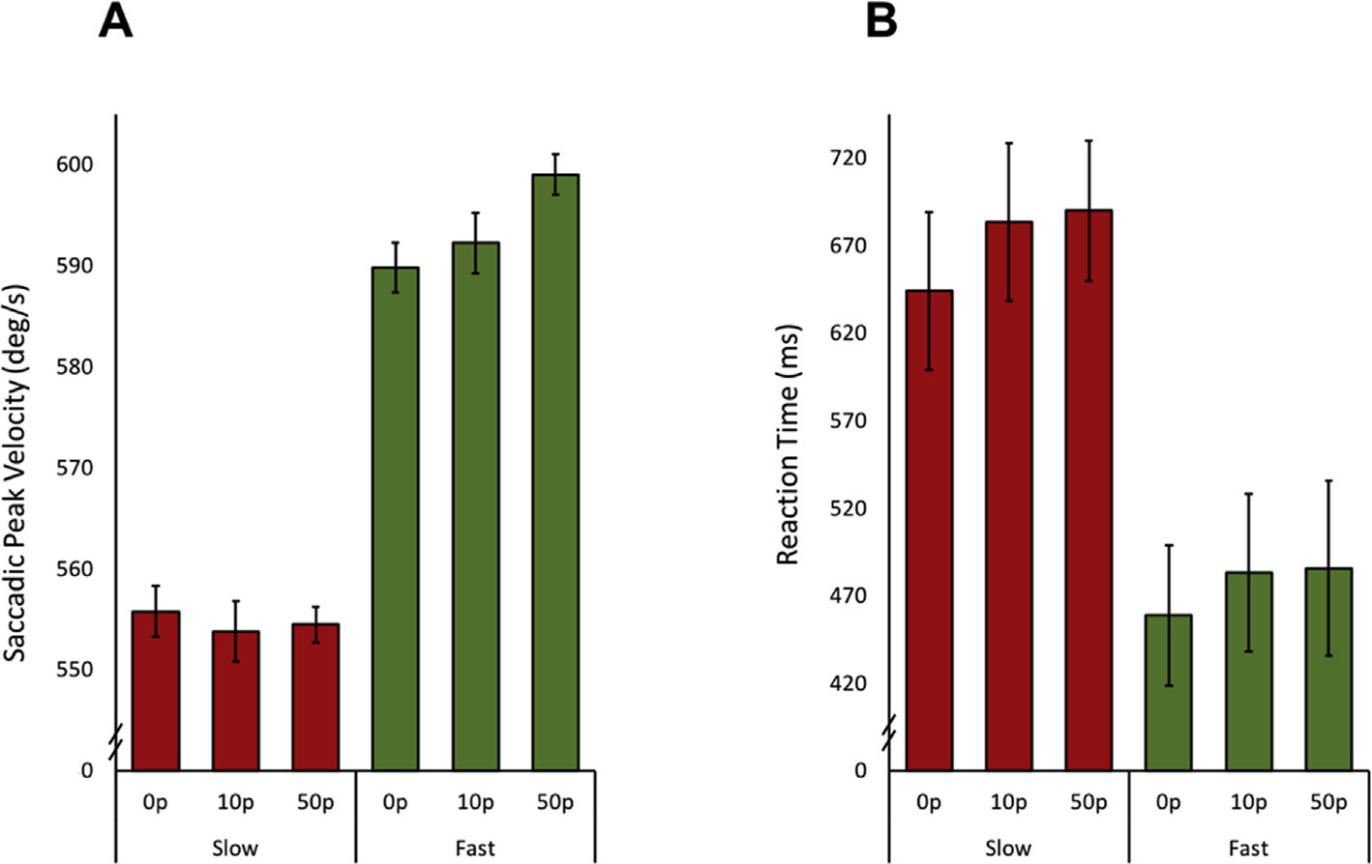
**Effect of loss on oculomotor properties in slow and fast saccadic velocity conditions. A.** Average saccadic velocity was significantly different between slow and fast conditions, but no significant differences in velocity were found between loss levels for either slow or fast conditions. **B.** A significant difference between reaction times in the slow and fast conditions was present, but again no differences between loss level was observed.

Unlike rewards, when reducing monetary loss was used as an incentive to achieve required slow or fast saccades there was no significant differences between loss levels [main effect of loss F (2,30) = 2.63, *p* = .09]. There was replication of the main finding that saccadic velocity can be modulated voluntarily [main effect of velocity condition, F (1,15) = 81.18, *p* < .000001]. However, no significant differences were attributable to the potential loss of not performing the required velocity [velocity × loss interaction F (2,30) = 2.47, *p* = .10; Fig. 8A].

To account for any non-sphericity in the data, where appropriate, statistics are reported with Greenhouse-Geisser correction. There were no differences in saccadic amplitude [main effect of velocity F (1,15) = .50, *p* = .49; main effect of loss F (1.42,21.22) = 1.46, *p* = .25; interaction F (1.83,27.49) = 1.01, *p* = .37] or amplitude variability [main effect of velocity F (1,15) = .0003; *p* = .99, main effect of loss F (2,30) = 1.17, *p* = .33; interaction F (2,30) = .69, *p* = .51]. Reaction time was also not influenced by loss level. However, as in the previous two experiments, reaction times in the fast saccade conditions were significantly shorter than for the slow condition [main effect of velocity F (1,15) = 5.41, *p* = .04; main effect of loss F (2,30) = 2.49, *p* = .10; interaction F (2,30) = .35, *p* = .70; Fig. 8B]. With the addition of loss in this experiment, there were no significant correlations between saccadic peak velocity and the LARS-e.

#### 3.5.2 Pupillary differences in preparation for fast and slow saccades to avoid loss

**Fig. 9:**
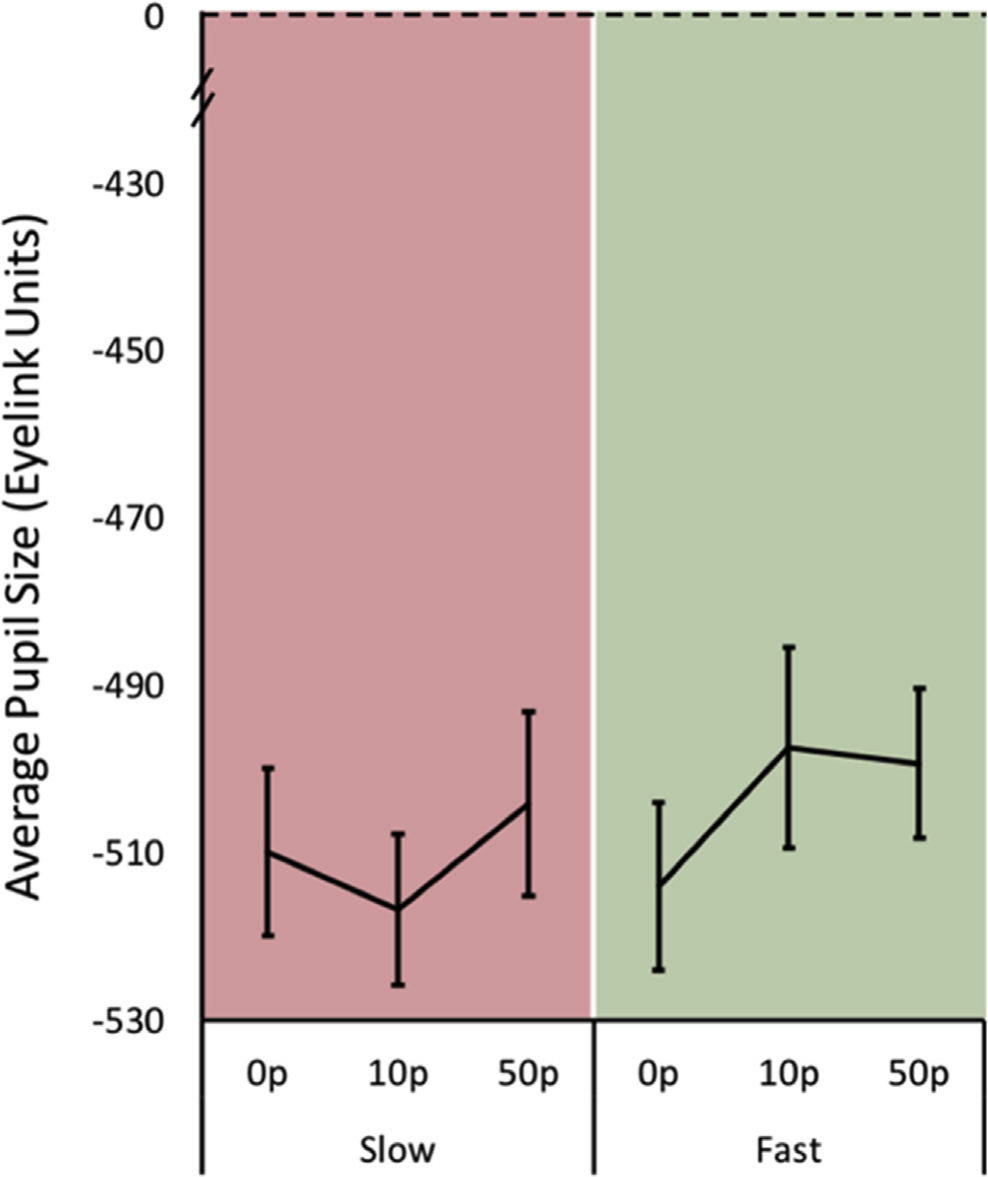
**Pupillary response to loss in the slow and fast velocity conditions.** No significant differences between fast or slow saccade conditions were observed, nor any differences between loss levels. Average pupil size in Eyelink units over 500 msec–1200 msec after fixation cross onset.

Unlike the reward experiment (Study Two), there were no differences in pupil modulation between loss levels when preparing to make either fast or slow saccades [main effect of velocity F (1,15) = .43, *p* = .52; main effect of loss F (2,30) = .66, *p* = .52; interaction F (2,30) = .50, *p* = .61; Fig. 9].

#### 3.5.3 Performance analysis across three studies

**Fig. 10:**
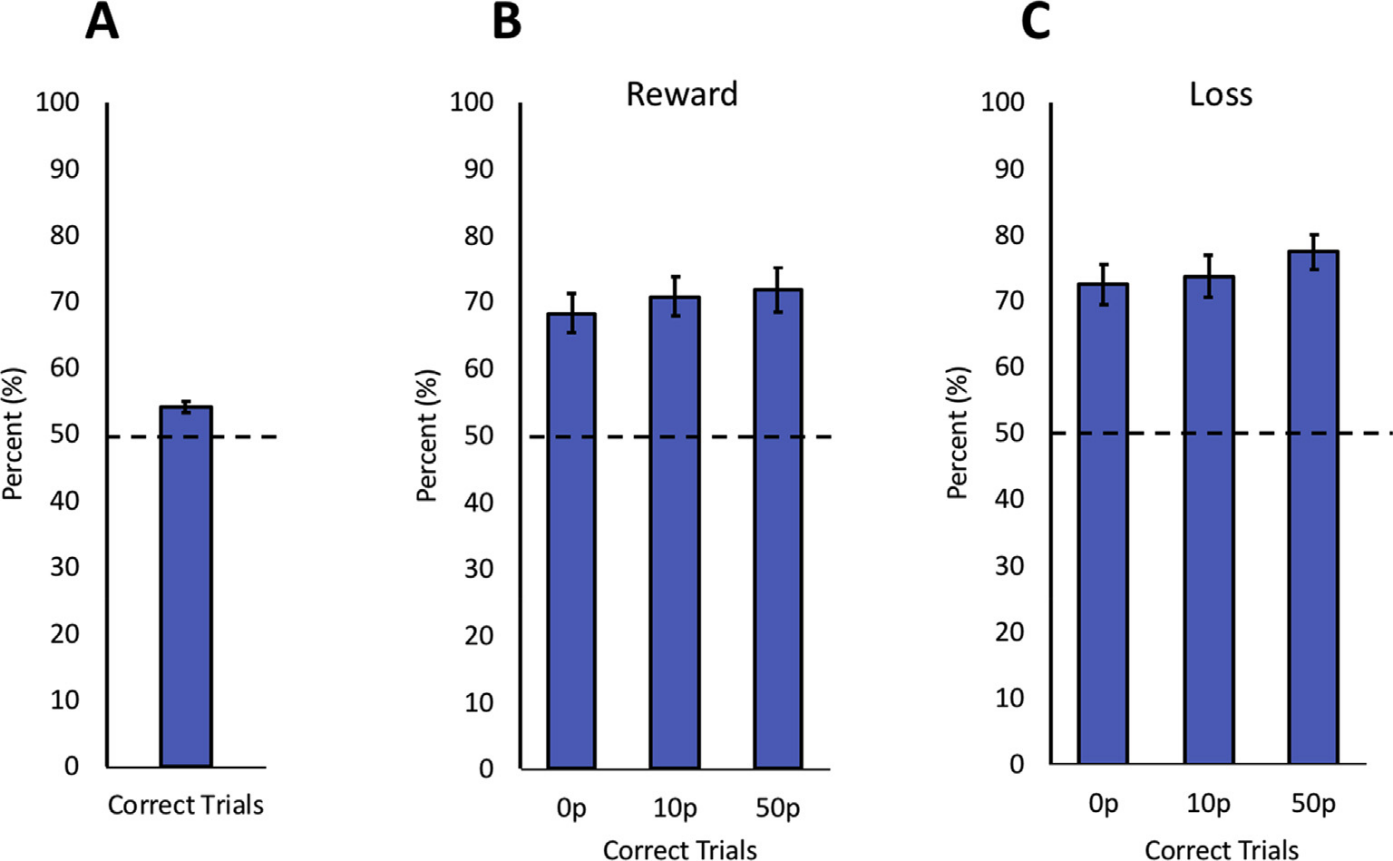
**Proportion of correctly modulated trials for no incentive, rewards and to avoid losses. A.** Percentage of successful trials in Study One where saccadic peak velocity was modulated appropriately, i.e., when instructed to go slow a saccade slower than the individual's baseline level was achieved and vice versa for the fast condition. This was significantly greater than chance, indicated by the dashed line. **B.** Percentage of successful trials where saccadic peak velocity was modulated appropriately across all reward levels (Study Two). **C.** Percentage of successful trials where saccadic peak velocity was modulated appropriately across all loss levels (Study Three).

Across all three experiments, participants were able to increase or reduce their saccadic peak velocity greater than expected by chance. In Study One (voluntary adaptation of saccadic velocity without any monetary incentive), on average 54.1% of trials across participants were accurate, i.e., they matched the condition type indicated by the white arrow at the start of each trial such that when a slow trial was required, saccadic velocity was less than the individual's baseline saccadic velocity, and for fast trials it was greater (*p* < .001; Fig. 10A). It should be noted that there was variability between subjects (accuracies ranged from 50% to 63%), and that people who scored higher on the LARS-e motivation questionnaire were more able, or indeed willing, to voluntarily change their saccadic velocities (Fig. 4B).

Importantly, the addition of incentives appeared to increase the ability to modulate velocity further. The proportion of trials where the required saccadic velocity was correctly produced appeared to increase for larger rewards (Study Two; Fig. 10B) or with the risk of increased losses being incurred (Study Three; Fig. 10C) compared to Study One where no monetary rewards or losses were involved. In Study Two, the mean proportion of correct trials for the 0p condition was 68.3%, for 10p reward trials was 70.8% and for 50p reward was 71.9%. In Study Three, which used loss as an incentive, the mean proportion of correct trials for the 0p loss condition was 72.6%, for 10p loss was 73.7% and for 50p loss was 77.4%.

#### 3.5.4 Comparisons across and between three studies

Peak velocity and reaction time correlations were examined across all subjects in all 3 experiments (N = 53). Participants with faster peak velocity (in either the slow or fast condition) did not have faster reaction times. However, those who were able to strongly modulate their velocity also modulated their reaction times. Within-subject trial-to-trial relationship of reaction time with peak velocity was also examined. Trials with shorter reaction times did have faster peak velocity; however, when comparing the relationships separately for ‘fast’ and ‘slow’ instruction trials, these did not overlap. So although reaction times reliably influences velocity, and reaction time is modulated by the instruction, the instruction influences velocity over and above what is expected from the change in reaction time alone. Thus, the reaction time was not confounding the velocity effect (See Supplementary materials Figure S4 for further details).

In experiment one, baseline peak velocity did not correlate with the ability to modulate saccadic peak velocity nor with overall LARS-e scores. This was also performed across all subjects in all 3 experiments to maximise power. In particular baseline peak velocity did not correlate with LARS-e measures of motivation (baseline r^2^ = .00699, *p* = .560). Furthermore, the index of modulation (peak velocity for fast minus slow instruction trials) was uncorrelated with baseline peak velocity (baseline r^2^ = .0500, *p* = .115).

In experiments two and three, there were significant correlations between average pupil size (time relative to the onset of the incentive cue) and saccadic peak velocity. Trials with larger pupil diameter had higher peak velocity. However this correlation was a steady contribution to pupil size, rather than being a phasic effect. There was no correlation between reaction time and pupil size. This would be in keeping with motor vigour mediated through arousal levels (See Supplementary materials Figure S6 for further details).

Finally, pupil responses were significantly different between experiment two and three over the 700 msec – 1200 msec window. There was a significant difference in the absolute global modulation of the pupil, *p* = .0030, t (30) = 3.23, SD 339.82, but no significant difference in the pupil baseline between reward and loss groups at time 0 msec (start of cue), *p* = .44.

## 4 Discussion

The findings presented here reveal that saccadic velocity, at least to some extent, can be modulated voluntarily, thereby breaking the stereotyped main sequence relating saccade velocity to movement amplitude (Bahill et al., 1975) (Fig. 4A). The ability to voluntarily control velocity was associated with individuals' motivation level: participants with higher scores on the LARS-e motivation questionnaire were more able to modulate their saccadic velocity (Fig. 4B). The addition of monetary rewards and losses enhanced participants’ ability to voluntarily control saccades further (Figs. 6A and 8A). Autonomic pupillary dilatation was greatest for larger rewards but not losses (Figs. 7A and 9) and incentives also increased the percentage of trials where saccadic peak velocity was appropriately modulated (Fig. 10).

The findings from this study suggest that saccadic peak velocity can be modulated by incentives on offer, but crucially that they can also be modulated through voluntary control. However, an alternative possibility is that changes in saccadic *amplitude* actually led to alterations in velocity. Given that peak velocity scales with saccade amplitude (Bahill et al., 1975), it is possible that when participants attempt to go faster, they are in fact just increasing the amplitude of their saccades and reducing amplitude for slower saccades. To address this, the effect of the main sequence on saccadic peak velocity was factored out: residual velocities for each saccade were calculated by regressing out the effect of saccade amplitude on peak velocity for each trial (Fig. 5C). The ability to modulate saccadic peak velocity still remained even after factoring out any effect of amplitude, confirming that modulation in velocity was possible.

Although previous investigations have assessed the effect of rewarding and aversive stimuli on saccades (Chen et al., 2014, Manohar et al., 2015, Manohar and Husain, 2016), this study is the first to our knowledge that explored whether saccadic velocity can be voluntarily modulated. Saccades provide a relatively pure measure of action with reduced degrees of freedom compared to limb movements, thus providing an important model system to study behaviour (Carpenter, 2004). But how is it possible that voluntary control and incentives alter saccadic velocity? Midbrain and pontine burst neurons linked to the generation of saccades are considered to play a key role in the control of their velocity (Sparks, 2002). These neurons receive afferents from striatal neurons, which are sensitive to expected incentives as demonstrated by neural recordings (Hikosaka, 2007). Frontal, parietal and striatal areas may also play a role in stimulating saccadic centres such as the superior colliculus, potentially allowing saccades to be voluntarily controlled and invigorating rewarded saccades (Munoz and Everling, 2004, Pouget, 2015). However, precise details of the underlying mechanisms remain to be elucidated.

Individual differences in motivation level in this study correlated significantly with the ability to voluntarily modulate saccadic velocity (Fig. 4B). The self-driven nature of voluntary saccades may explain this finding. Inherent value of visual information has in itself been shown to influence motor commands for saccades made in anticipation of viewing faces rather than random pixels (Xu-Wilson, Zee, & Shadmehr, 2009). Self-driven unrewarded saccades may also reflect this *intrinsic* drive. By contrast, externally incentivized movements might more likely be related to *extrinsic* drive, an aspect of motivation that the questionnaire scores may not capture. Hence the lack of association with the LARS-e motivation scores when incentives were included. The undermining effect of external incentives on intrinsic motivation has been shown in several behavioural studies when monetary rewards were actually found to hamper performance (Deci, 1971). Intrinsic drive is the behaviour to obtain an outcome that is inherently important to an individual, providing satisfaction that is not in return for other consequence such as receipt of a reward (Ryan & Deci, 2000). The intrinsic value of being able to correctly control saccadic velocity, even when no monetary incentives were available, may account for the significant association with motivation observed in this study.

For experiment one, average baseline peak velocity taken at the start of the experiment and the average velocities produced during the course of the experiment did differ (Figure S1). This means that online feedback did not always accurately reflect performance as baseline trials were used for determining displayed outcomes at the end of each trial. However we did not expect the feedback to influence performance in this task with respect to whether participants were able to modulate their velocity on instruction. Indeed participants’ ability to modulate saccade peak velocity was still present in individuals who had slower or faster baselines compared to average velocities during the course of the experiment. Although it could be argued that variability in feedback may influence performance, as demonstrated in our results subjects could still modulate their peak velocity and produce faster or slower saccades when instructed. This is the key result we emphasize. To ensure this could not bias the results in experiments two and three, an adaptive baseline was used.

Saccadic peak velocity increased with the addition of both rewarding and aversive incentives, but only when fast saccades were required to be made. There were no significant differences between incentive levels for slower saccades (Figs. 6A and 8A). This result may signify a floor effect in the slow condition: reducing saccadic velocity further even with the addition of incentives might not be possible as there may be a minimum velocity produced by burst neurones in order to generate a saccade (Leigh & Kennard, 2004). In addition, incentives are known to invigorate motor responses (Manohar et al., 2015) but when coupled with the instruction to perform a slow saccade, the outcome may be that each factor counteracts the other. Hence, the invigorating effect of incentives may be suppressed by actively trying to make slower saccades. The three studies were adequately powered to detect the effect of fast versus slow (sample size estimated from experiment one is N = 3). It is possible however, that experiment three was underpowered to detect an effect of penalty. A previous experiment in humans also failed to find effects of penalty expectation (Manohar, Finzi, Drew, & Husain, 2017), however this was a vigour task not investigating voluntary modulation. Further, In a monkey study of eye movements and incentives, saccade peak velocities were highest for rewarded trials, lowest for punishment and in between for neutral conditions. This suggest that saccade velocity may be a reflection of subjective value and not motivational salience (Kobayashi et al., 2006) possibly accounting for why no significant differences in velocity were found between degrees of loss. Punishments therefore, may have less of an effect on saccadic modulation due to reduced valence compared to rewards. Although the presence of monetary rewards or losses significantly increased the ability to correctly modulate saccadic velocity compared to when no incentives were provided, each study was performed by a different group of participants so these conclusions have to be moderated by the possibility of overall group differences.

In addition to eye movements, assessment of physiological responses through autonomic modulation of pupil size has been influential in increasing our understanding of goal directed behaviour and providing an objective assessment of motivation level. Again, neurons in the superior colliculus appear to play a key role in evoking transient pupil dilation, in addition to being associated with preparatory processes in the generation of saccades (Wang, Brien, & Munoz, 2015). Top-down inputs from the fronto-parietal cortex, locus coeruleus and basal ganglia are able to alter pupil response in reward-related cognitive tasks (Wang & Munoz, 2015). These effects may, in part, underlie the increase in pupil responses for larger monetary rewards – but not losses – observed in the present study (Fig. 7, Fig. 9).

Pupillary responses have also been used to index dysfunction of reward processing in patients who suffer from disorders of motivation, for example in conditions such as clinical apathy. Reward-evoked pupil responses are blunted in Parkinson's disease patients who lack motivation (Muhammed et al., 2016). Pupillary modulation by extrinsic incentives may therefore be reflective of motivational abnormalities and also index the dopaminergic reward system. Such a possibility is given support by the demonstration that pupil responses to reward are reduced in Parkinson's disease patients when off dopamine medication (Manohar & Husain, 2015). Despite the association with motivation in previous studies, the current work did not find links between motivation questionnaire scores and pupillary changes in healthy young people. This may be because the tasks also included saccadic voluntary control, a factor not explored before. Control of eye movement velocity is normally an automatic process that is not consciously controlled. Voluntary modulation of saccadic motor speed requires effort. On informal debrief after performing the experiments, the majority of participants commented that modulating saccadic velocities felt effortful. Allocating effort to modulate and energize actions has been shown to modulate pupil size (Varazzani, San-Galli, Gilardeau, & Bouret, 2015). Consequently, differences in pupil response in the current study may not only be linked to *incentive evaluation* associated with motivation, but also reflect *effort allocation*. This factor might account for the lack of correlation between individual differences in motivation and pupil response.

Overall pupil responses were significantly different between experiment two and three over the 700 msec – 1200 msec specified window. Pupil size in the loss condition were smaller compared to reward. However, factors of interest were within-subject pupillary differences, not absolute responses or quantitative differences between the studies. One interpretation of the difference between studies could be that the loss context causes less dilatation compared to responses in the reward experiment. This was not explained by different baseline pupil sizes in the two studies. The luminance was also controlled across studies and therefore should not be the cause of the difference either. It may instead be due to heterogeneity in the groups as different subjects participated in each experiment, or indeed the effect of the penalty context. Manohar et al. demonstrated that loss has reduced effects on pupil modulation compared to rewards (Manohar et al., 2017) which may explain our findings. Given the current design, it is not possible to disentangle this further, as we do not have reward and loss in the same participants.

In conclusion, the findings here show that healthy young participants were able to modulate the velocity of their saccades voluntarily to some extent. This ability was associated with individual differences in motivation level and was further modulated by the addition of monetary rewards and losses, breaking the stereotyped main sequence.
