# Supplementary material for: Voluntary modulation of saccadic peak velocity associated with individual differences in motivation
Source: Cortex. 2020 Jan;122:198–212. doi: 10.1016/j.cortex.2018.12.001 (PMC6970223; doi:10.1016/j.cortex.2018.12.001)
Supplement: Multimedia component 1 [file mmc1.docx]

**Supplementary Materials**

**Voluntary modulation of saccadic peak velocity associated with individual differences in motivation**

K. Muhammed, E. Dalmaijer, S. Manohar & M. Husain.

**Methods:**

**Further experimental paradigm details:**

In Experiment two and three, The reward levels were given via spoken words, these were matched for volume and duration. In experiment two, a "Ka-ching!" cash register noise was played at the end of a trial for successfully receiving a reward, regardless of level. This was provided after the completion of each trial so as not to influence anticipatory pupil changes or other oculomotor properties. For poor performance in experiment three, when participants lost money and for all levels of loss, a sine wave (frequency 440 Hz, length 200ms) was played. The tones in each experiment were matched for volume and duration.

In all studies, the fixation cross had a diameter of 0.5 degrees of visual angle and the target was 0.78 degrees of visual angle.

The average peak velocity of the previous trials in a condition without any weighting was taken. e.g. in experiment two and three the average of the previous 20 "slow" trials were used as the threshold for the current "fast" trial.

Selection criteria was also applied to reaction time, trials with a latency of less than 90ms and greater than 3000ms were rejected. In total, approximately 5% of trials were disregarded.

Blinks shorter than 500ms were interpolated using linear segments after discarding samples before and after the blink where the slope of the pupil trace was steeper than 2.5 AU/ms. This is the same method as used in (Manohar & Husain, 2015).

Payment was provided to participants for all 3 experiments. In experiment one, the amount obtained was fixed between £8-12 with a minimum given of £8. At the end of the experiment the exact amount taken away by a participant was calculated as the percentage of correct trials performed as a proportion of £4 which was then added to the minimum £8 payment.

For experiments two and three a similar scaling procedure was implemented to calculate the payment obtained.

This was based on overall correct trials, regardless of reward or loss level. The overall proportion of correct trials over the course of the experiment was used to calculate how much of the £4 would be added to the minimum earnings of £8 in the reward experiment and subtracted from £12 in the loss experiment.

Participants were not told how the final rewards were calculated, only that it was performance dependent, so the better they performed the more they would receive in experiment two, or not lose in experiment three.

**Results:**

**FIGURE S1**


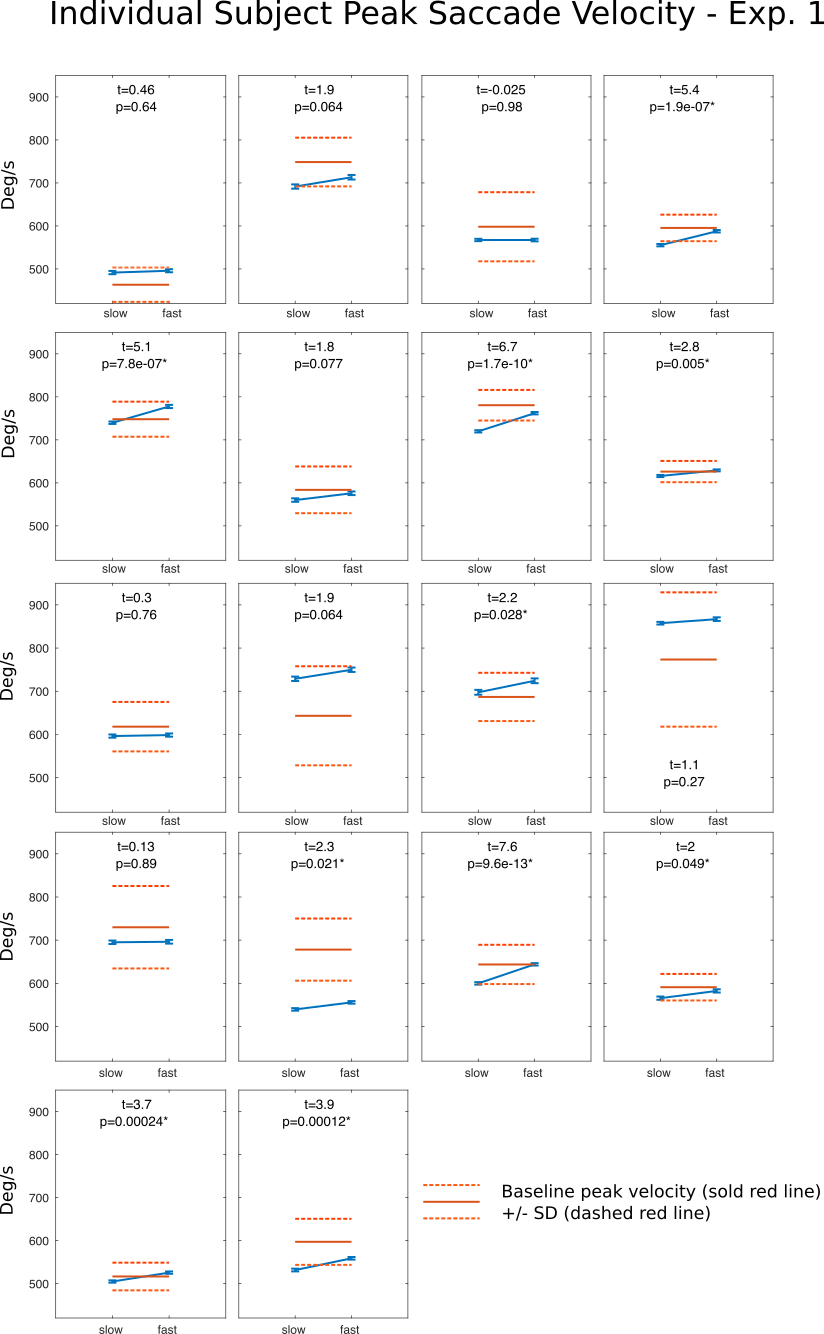


**Fig. S1:** **Per-subject peak velocity in experiment one**. The mean peak velocity and within-subject standard error for the fast and slow saccades are shown in blue. Red lines indicate the mean and standard deviation of the velocities measured during the baseline trials. An individual’s ability to modulate saccadic peak velocity between the slow and fast condition is shown by the p-value for each subject.

**FIGURE S2**

**
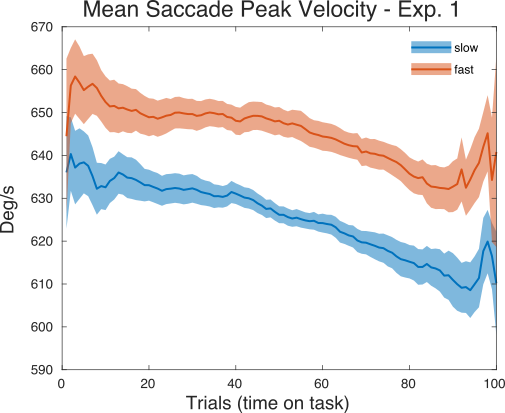
**

**Fig. S2:** **Fatigue effect on saccadic peak velocity in experiment one**. The trend of peak saccade velocity across the task is shown using a sliding time-window. The line indicates the mean and shaded area indicates standard error across subjects. Means were calculated over the trials, with the trials filtered by condition (fast versus slow). A significant effect of fatigue was found but this did not alter participants ability to modulate saccade velocity.

The possibility of systematic trial-to-trial changes in velocity are controlled for in studies two and three. However, in study one this was not the case. Therefore the effect of fatigue over the course of the experiment may have influenced the feedback that participants received potentially impacting on the results. To address this, a running average of peak velocity of all participants, over the duration of the experiment was assessed. To test if the effects of fatigue were important in experiment one, we binned trials into early (first half of experiment) and late (last half), and performed a 2x2 rmANOVA on peak velocity. There were main effects of the speed cue (F(1,68)=24.92, p<0.001) and time-on-task (F(1,68)=5.26, p=0.025), but no interaction (F(1,68)=0.229, p=0.63). This indicates that although participants fatigued, it did not alter their ability to modulate saccade velocity.

**FIGURE S3**

**
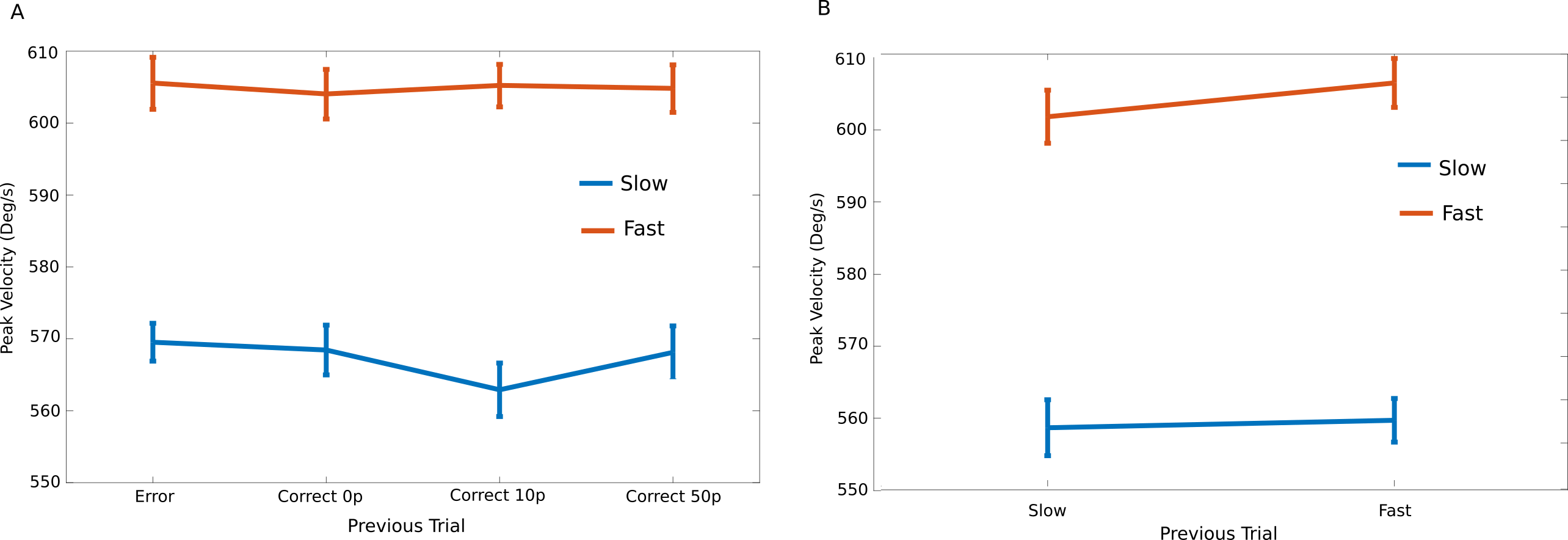
**

**Fig. S3:** **Saccadic peak velocity trial to trial effects**. (A) Trials in experiment two were split according to the previous trial’s outcome. Trials could be error trials where no reward was obtained, or could be successful in which case either 0p, 10p or 50p was obtained. The mean and within subject standard error of average peak velocity in each group of trials is shown. There was no effect of the previous trial in both fast or slow conditions. Mean peak velocity for trials in the slow condition in degrees per second; error trials; 569.5, correct 0p; 568.4, correct 10p; 562.9 and correct 50p; 568.1. For the fast condition error trials; 605.5, correct 0p; 604.0, correct 10p; 605.2 and correct 50p; 604.8. The same findings were also true in experiment three (not shown). (B) Trials were split in experiment two according to whether the instruction on the previous trial was to go fast or slow. There was no effect of the previous trial type (speed cue) on peak velocity. Mean peak velocity in degrees per second for the slow condition when the previous trial type was slow; 556.8, and when the previous trial type was fast; 557.6. Mean peak velocity in the fast condition when the previous trial type was slow; 602.7 and when the previous trial type was fast; 606.7.

**FIGURE S4**


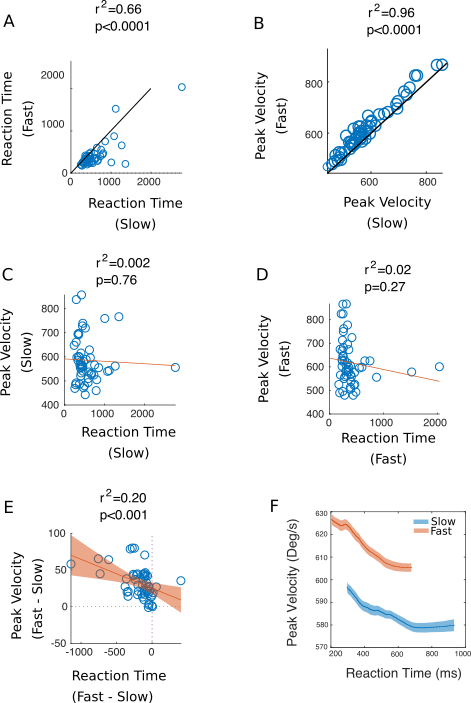


**Fig. S4:** **Comparison of average oculomotor measures across all subjects in all 3 experiments (N=53)**.

(A) Participants with faster reaction times (RT) in the fast condition, also have faster RT in the slow condition. Most people fall below the 1-to-1-line indicating that RT was faster in the fast condition than in the slow condition.

(B) Subjects with fast peak velocity (PV) in the fast condition also had fast PV in the slow condition.

(C) No correlation was present between PV in the slow condition and RT in the slow condition. Subjects with faster PV in the slow condition did not have faster RT.

(D) No correlation was found between PV in the fast condition and RT in the fast condition. Those with faster PV in the fast condition did not have faster RT.

(E) Subjects who strongly modulated peak velocity also strongly modulated RT.

(F) Within subjects analysis of binned RT for each condition by quantiles using an overlapping window of 20% of trials and mean PV in each RT bin. For each bin this is shown as mean +/- SEM.

Peak velocity and reaction time correlations were examined across all subjects in all 3 experiments (N=53). Firstly, participants who had faster reaction times (RT) in the fast condition, also had faster reaction times in the slow condition. Notably most people fell below the 1-to-1-line indicating that their reaction time was faster in the fast condition than in the slow condition (**Fig. S4A**). Similarly, people with fast peak velocity (PV) in the fast condition also had fast PV in the slow condition. All but 1 participant fell above the 1-to-1 line indicating that their velocities were faster in the fast condition than the slow condition. (**Fig. S4B**). Interestingly, subjects with faster PV (in either condition) did not have faster RT (**Fig. S4C and D**). However, those who were able to strongly modulate their velocity also modulated their RT (r^2^=0.20 p<0.001) (**Fig. S4E**). Within subjects, RT was binned for each condition by quantiles (overlapping windows of 20% of trials), and the mean PV in each RT bin was calculated. This quantiled analysis corrected for between-subject variability in overall speeds. For each bin, velocity is shown as mean +/- SEM. across subjects. (**Fig. S4F**). RTs were faster in the fast condition (left-shift), and PV was faster in the fast condition (upward-shift), as seen previously in panels A and B. However, there was a strong correlation across trials between the PV and RT, even within a condition. So trials that were initiated quickly, also tended to have higher PV. The velocity effect could not completely be accounted for by the RT speeding as indicated by the lines not being superimposed (a vertical shift between the lines). Thus, the RT is not confounding the velocity effect. Moreover, the fast versus slow effect is seen for even the shortest-latency trials, suggesting that instructions can even modulate relatively reflexive movements.

**FIGURE S5**


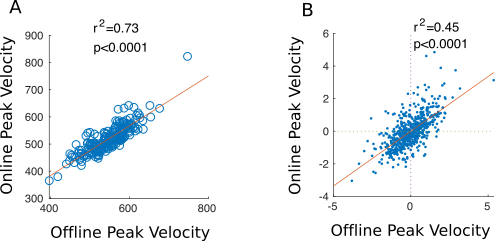


**Fig. S5:** **Average online calculation of peak velocity versus offline peak velocity calculations**.

(A) An example of online versus offline peak velocity from an single participant in experiment one demonstrating the strong correlation between calculations. (B) Z-scored online velocity estimates correlated very strongly with offline estimates across all participants in experiment one, the online peak velocity measures were used to give the live feedback on performance at the end of each trial.

Online estimates of peak velocity for each trial were calculated using the algorithm described in Dalmaijer et al, which uses point-to-point differences in space and time to compute velocity, and computes acceleration over three samples, as the difference in velocity (Dalmaijer, Mathôt, & Van der Stigchel, 2014). This algorithm was designed for gaze-contingent interfaces, and we used it only for the online detection of saccades. Upon the detection of a saccade, it was tested whether its starting point had been within 2 degrees of the fixation cross’ centre, and its endpoint within 5 degrees of the target’s centre. Peak saccade velocity was defined as the highest value of the pointwise division of all inter-sample distances by all inter-sample times.

Offline precise measurements of saccadic velocity were used for all subsequent analysis. This used standard Eyelink criteria for detecting saccades motion, velocity, and acceleration. Saccade velocity threshold was set to 30 degrees/second, saccade acceleration threshold was set to 8000 degrees per second and saccade motion threshold 0.15 degrees per second^2^. Peak velocity was estimated as in (Manohar et al. 2015, 2017) from the raw position trace by calculating the magnitude of the velocity vector at each millisecond sample, taking an average over 3ms windows during the saccade, and finding the maximum

**FIGURE S6 A and B.**


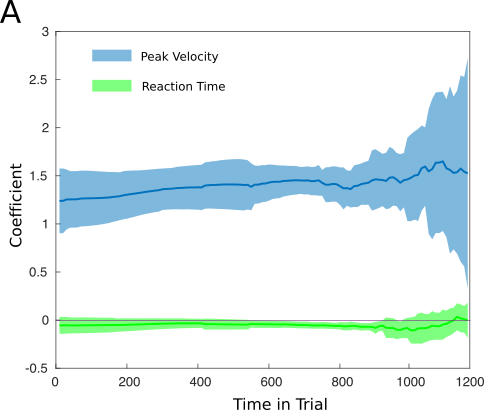

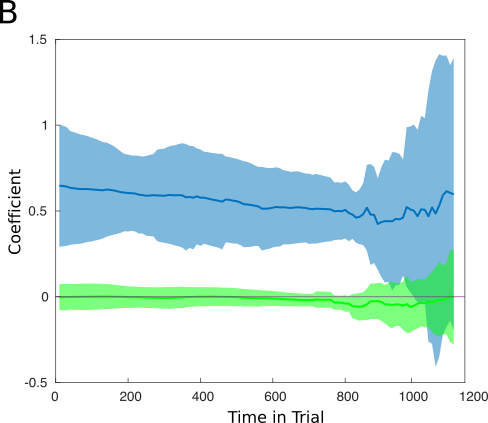


**Fig.S6:** **The correlation between pupil size, and the peak velocity and reaction time on each trial**. Normalised regression coefficients are shown as a function of time after the cue. Shaded area is standard error across participants. Faster velocities were associated with larger pupil sizes (blue), but these seemed related to the tonic pupil size rather than the phasic response to the cue. There was no relation with reaction time (green). This was true both when reward in experiment two (A) and loss in experiment three (B) were examined.

**FIGURE S7**

**
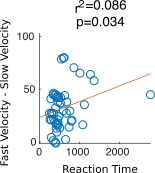
**

**Fig. S7:** **The difference between peak velocity in the fast condition and peak velocity in the slow condition in experiment one across subjects, as a function of reaction time.**

Participants who had a longer reaction times were able to modulate their saccadic velocities to a greater extent.

A significant correlation was present across all experiments between participants ability to modulate saccadic peak velocity (fast peak velocity – slow peak velocity) as a function of reaction time. R^2^ = 0.07, p=0.04.

In all experiments, the reaction times in the ‘baseline’ conditions were much faster than the ‘slow’ conditions. There were no significant differences between baseline and the fast condition. This would suggest that participants are actively waiting in the “slow” condition.

| **RT** | Baseline | Fast | Slow | Baseline < Fast | Baseline < Slow |
| --- | --- | --- | --- | --- | --- |
| **Exp1** | 287 (20.0) | 490 (46.3) | 369 (37.4) | t(17)=2.03, p=0.058 | t(17)=4.21, p<0.001 |
| **Exp2** | 416 (33.5) | 374 (34.8) | 592 (70.0) | t(18)=-1.11, p=0.28 | t(18)=3.48, p=0.0027 |
| **Exp3** | 406 (46.5) | 477 (131) | 673 (158) | t(15)=0.63, p=0.54 | t(15)=1.96, p=0.069 |

RT = Reaction Time
(Standard deviation)

**Discussion:**

Part of the effort in performing the tasks was in slowing saccadic peak velocity. Participants subjectively felt this was hard to achieve, so it may be plausible that more reflexive saccades produced in gap tasks, (where the fixation cross would disappear prior to the onset of the target) participants may lose the ability to slow down velocity. To address this in our data, we examined potentially ‘more reflexive’ trials by binning by reaction time (see **Fig. S4F**). We can examine velocity modulation by the velocity (vertical) distance between the ‘fast’ and ‘slow’ instruction conditions. When trials are binned by reaction time, we do not find that the modulation is lower for the short reaction time trials. This suggests that speed might be modulated even when saccades are more reflexive.
